# Association between Endocrine Disrupting Phenols in Colostrums and Maternal and Infant Health

**DOI:** 10.1155/2013/282381

**Published:** 2013-05-08

**Authors:** B. Yi, C. Kim, M. Park, Y. Han, J. Y. Park, M. Yang

**Affiliations:** ^1^Research Center for Cell Fate Control, Sookmyung Women's University College of Pharmacy, Seoul 140-742, Republic of Korea; ^2^Korea Testing & Research Institute, Gyeonggido 415-871, Republic of Korea; ^3^Inje University Sanggye Paik Hospital, Seoul 139-707, Republic of Korea; ^4^Mart Pediatric Clinic, Gyeonggido 456-824, Republic of Korea; ^5^Division of Cancer Prevention and Controls, Moffitt Cancer Center and Research Institute, 12902 Magnolia Drive, Tampa, FL 33612, USA

## Abstract

Bisphenol A (BPA) and alkylphenols (APs) are well-known endocrine disrupting chemicals (EDCs) which may threat the next generations' health. We performed biomonitoring of these phenols in colostrums to assess risk of the phenols in breast-fed neonates. Study subjects were the lactating mothers who delivered babies within 2 weeks (*N* = 325; 30.67 ± 3.45 years) and their neonates (*N* = 326; embryonic period, 39.1 ± 1.5 weeks). BPA, nonylphenol (NP), and octylphenol (OP) in colostrums were quantified with LC/MS/MS. Information for environmental exposure sources of the phenols was obtained by questionnaires. As results, median level of BPA in colostrums was 7.8 ng/mL, while most NP or OP was not detected. Regarding health risks of phenols, levels of total NP in colostrums were elevated among sick mothers with toxemia, thyroid disorders, gastritis, and so forth than health mothers (3.51 ± 4.98 versus 2.04 ± 3.71 ng/mL, *P* = 0.02). Dairy products intake and detergents use were positively correlated with total BPA levels (*Ps* < 0.05). In conclusion, we estimate most neonates who are exposed to BPA rather than NP or OP via colostrums and recommend continuous biomonitoring of the phenols to clarify their suspected health risk on neonates and pregnant or gestation mothers.

## 1. Introduction

Effects of endocrine disrupting chemicals (EDCs) on human health and wildlife are receiving growing attention for the next generation's health and have been known to interfere with endocrine systems by mimicking, blocking, and triggering actions of hormones and implicated with toxic effects, for example, disorders in development and reproduction [1]. Among EDCs, bisphenol A [BPA, 2,2-bis (4-hydroxyphenyl) propane] is widely used for a variety of applications, for example, baby feeding bottles, food-can lining, and sealants in dentistry. In addition, 4-tertiary-octylphenol (OP) and 4-nonylphenol (NP) of alkylphenols (APs) have been used to make alkylphenol ethoxylates, nonionic surfactants applied as emulsifying, wetting, or stabilizing agents in industries, and various consumer products including detergents and pesticide formulations [2]. Due to the wide uses of these phenols, it has been speculated that human exposures to environmental phenols may be widespread [3–6]. Thus, concerns about various adverse health effects caused by EDCs are increasing, and rigid risk assessment for EDCs throughout valid biomonitoring studies has been called for. Particularly, considering low-body weights and susceptibility, we suspect that body burden or real exposure level of infants or children to BPA or APs is expected to be heavier than those of adults. In the view of susceptibility, the exposures to environmental phenols in infants and children have got the public attention because EDCs treat the second generation's health, for example, genital malformations, testicular abnormalities, impairment in fertility or sexual functions, and neonates are considered to be a vulnerable subgroup to xenobiotics [7, 8]. Therefore, environmental phenols including BPA and APs should be continuously monitored for achievements of public heath, particularly for infants and children. 

Contamination of ECDs in colostrums raises concerns gravely because neonates, who are solely dependent on colostrums, are considered to be a high susceptible to EDCs. Detoxifying enzymes of neonates would not be fully developed at this early time point and exposure to EDCs during the critical periods of developments could cause morphologic and functional alterations by influencing growth, reproduction, and development [7, 8]. Considering the characteristics of EDCs that affect the second generation's health, we need biomonitoring of EDCs in colostrum, which is the main route of exposure to EDCs for breast-fed infants. A number of investigations have reported the occurrence of several environmental chemicals such as persistent organic pollutants (POP), polychlorinated dibenzo-dioxins (PCDDs), and organochlorines (OCs) in breast milk [9–11]. BPA, OP, and NP have the potency to partition into breast milk, since they are lipophilic compounds, which have octanol-water partition coefficient value (log *P* or Kow) around 3-4 [12, 13]. 

Concerning the phenol exposure sources, we have studied various environmental sources; however, we could not find crucial exposure sources, yet [14, 15]. In a case of NP, dairy products and sea food were suspected as its exposure sources [16, 17]. In addition, ethoxylation products of APs have been used for cosmetics or surfactants [18]. Thus, we focused on the consumption of dairy products and sea food or the use of cosmetics or surfactants to find phenol exposure routes in this study. In addition, we established a sensitive analytical method for BPA, OP, and NP in human colostrums and performed biomonitoring of these phenols among Korean lactating women's colostrums to assess risk of BPA and APs for breast-fed neonates.

## 2. Materials and Methods

### 2.1. Study Subjects

Study subjects were 325 lactating mothers, who stayed in postpartum care centers in Seoul, Republic of Korea. All subjects consented to participate in this study and donated their colostrums (*≈*10 mL). Colostrums were collected in dark glass tubes with glass caps and stored −20°C prior to analyses. We also obtained their questionnaires addressing physical characteristics, lifestyle patterns including dietary habits, and health and pregnancy-related properties [14, 19]. All of the above procedures were approved by the institutional review boards of Inje University, Paek Hospital (Seoul, Republic of Korea). 

### 2.2. Analysis of Phenols in Milk

Throughout the entire procedure, plastic wares were replaced with glassware in order to avoid possible phenol contamination. We analyzed total forms (conjugated and free phenols) and free forms of BPA and APs for each colostrum sample with/without enzyme hydrolysis, respectively. The concentration of conjugated phenols was calculated after subtracting the amount of the free form of phenols from that of total phenols. 

To determine total forms of the three phenols levels in colostrum, we established an optimal method with modification of the BPA analysis. In brief, 100 uL of internal standard (125 ng/mL of bisphenol B (BPB), Tokyo Kasei Chemical, Tokyo, Japan), 120 uL of 2.0 M sodium acetate, and 48 uL of *β*-glucuronidase (2,784 U) (Sigma, St. Louis, MO) were added to 2 mL of milk samples. The mixture was incubated at 37°C for 5 hours; 4 mL of 2-propanol was added and mixed thoroughly. Thereafter, the mixture was centrifuged (3,000 g, 20 min), and 3 mL of supernatant was transferred to new glass tubes. This extraction was repeated, and total transferred 6 mL of supernatant was evaporated. After the evaporation, the residue was dissolved with 250 uL of 60% acetonitrile, and this solution was centrifuged (14,000 rpm, 10 min). Thereafter, 200 uL of the supernatant was transferred to a vial, and 50 uL of the supernatant was injected for LC/MS/MS analysis. The LC/MS/MS system was composed with Waters alliance 2695 XELC/MS/MS (Waters, Watford, UK) and Zobax SB-C18 (5 *μ*m, 4.6 × 250 mm, Agilent, USA). Separation was accomplished with a gradient mode: mobile phase A, water: mobile phase B, acetonitrile: flow rate 0.3 mL/min, and ratio of A to B: 0–3 min, 70 : 30; 3-4 min, from 70 : 30 to 95 : 5; 4–6 min, from 95 : 5 to 100 : 0; 6–25 min, 100 : 0; 25–30 min, from 100 : 0 to 70 : 30. The Waters alliance 2695 Quattro Premier XE was used in negative ion ESI mode. The electrospray ionization (ESI) settings were as follows: capillary voltage, 3.5 kV; cone voltage, 40 V; desolvation gas-flow (Argon gas), 800 L/hr; cone gas-flow, 20 L/hr; collision energy, 20 V. To determine concentrations of the free forms of phenols, we followed the procedures described above except enzyme hydrolysis.

### 2.3. Statistical Analyses

Distribution normality of the phenols levels was tested with Shapiro-Wilk *W* test. If their probability (*W*) is <0.05, their distribution is considered not to follow normal distribution. 

“Nondetectable” was assigned a value of half of the minimum value of detected each phenol level for further statistical analyses. Considering each value's characteristic (normality and nominal, continuous, or ordered values), Wilcoxon test, Fisher's exact test, Spearman's rho, and regression analysis were used for the study analyses. *P* < 0.05 was considered to be statistically significant. The statistical package of JMP Version 4 (SAS Institute, Cary, NC, USA) was used for all analyses. 

## 3. Results

### 3.1. Characteristics of Study Subjects

The characteristics of the subjects are summarized in Table 1. Mothers were 30.67 ± 3.45 years old, and, as expected, their body mass indices (BMIs) were increased during the pregnancy (median of before and after: 20.19 and 23.53 kg/m^2^, resp.). Based upon education level, occupation, and monthly household income, participants reflected typical middle class in the Republic of Korea. Approximately, 11% (*N* = 35) of the mother have clinical disease(s), such as toxemia (*N* = 22), thyroid disorders (*N* = 6), and gastritis (*N* = 1). Similar number of the infants were sick at birth (*N* = 34), and most of the sick babies were recovered within 2 weeks after birth or on the therapy except two infants who were born with a congenital malformation. There was no association of disease presence between the mothers and their infants (*P* = 0.66). 

### 3.2. Exposure Levels of the Three Phenols in Milk

We established a sensitive analytical method to measure BPA and APs in colostrum samples with LC/MS/MS. Limit of detection (LOD) and limit of quantification (LOQ) were calculated with signal to noise ratio 10 and 30, respectively. The calibration curve for simultaneous analyses of the three phenols was obtained within 5 different concentrations in phenols-spiked milk. The recoveries of BPA, NP, and OP were over 80%.

The distributions of the three phenols were left skewed near to zero, that is, nondetectable values and did not follow normal distributions (*Ps* < 0.01 by Shapiro-Wilk *W* test for nonnormality). Ranges of the three phenols' levels in colostrum were shown in Table 2. OP and NP were not detected in most colostrum samples as free forms without any metabolism. In addition, there was a significant positive correlation between total OP and NP levels (*β* = 0.37, *P* < 0.01 by regression analysis) and somewhat positive association between total OP and BPA levels (*β* = 0.06, *P* = 0.09).

Finally, we could estimate the daily exposure to the phenols in the infants via their mothers' milk and determined that the current exposure levels to BPA and NP were lower than their tolerable daily intakes (TDIs) (Table 2). 

### 3.3. Exposure Routes of the Phenols in Mothers

To identify the potential routes of phenols exposure from environment, we investigated associations between phenol levels in colostrum and exposure sources. We observed that dairy products intake and detergents use were positively correlated with total BPA levels (*P* < 0.05) (Table 3). However, we did not find any statistically significant association between phenol levels and other exposure (Table 3) including air pollution at resident area or use of plastic wrap. 

### 3.4. Effects of Phenol Exposures on Mother and Infant Health

Considering no associations between mothers' and infants' disease presence, we separately analyzed effects of phenol exposure on mothers or infants. At first, we analyzed effects of phenols exposure on physiological characteristics. Mothers' age was not associated any phenol levels; however, embryonic period in neonates showed somewhat negative association with NP levels (*β* = −0.25, *P* = 0.09). Concerning phenols disposition at adipose sites, we studied effects of phenol exposure on BMI. As result, we found some positive associations between levels of mothers BMI (before and after delivery) and NP (*β* = 0.09, *P* = 0.01 and *β* = 0.04, *P* = 0.19, resp.) or OP (*β* = 0.07, *P* = 0.07 and *β* = 0.03, *P* = 0.34, resp.). However, there were some negative associations between mothers BMI (before and after delivery) and levels of BPA (*β* = −0.11, *P* = 0.07 and *β* = −0.09, *P* = 0.12, resp.). 

Secondly, we found that levels of total NP in mothers or infants with diseases were higher than those in health subjects (Table 4). In the case of mothers' toxemia, a major mother disease (*N* = 22), we found more strong association between the disease and NP exposure (*P* < 0.01). For neonates' major disease, jaundice (*N* = 13 including recovered cases), we could not find any significant association between the disease and the 3 phenol levels (*Ps* > 0.05). In addition, neonates with congenital malformation (*N* = 2) showed somewhat high levels of BPA, NP, and OP rather than others (*P* = 0.07, 0.11, and 0.27, resp.). In detail, the colostrum of the infant with congenital malformation of uvulas showed quite high levels of total NP and OP (medians, 21.5 and 18.1 ng/mL, resp.) compared to others' levels (medians <LOD, i.e., <0.5 and <0.3 ng/mL, resp.). 

## 4. Discussion

Considering physical complexity as a matrix of colostrum, proper preparations of colostrums specimens are required. Recently, many researchers have used solid phase extraction (SPE) for this purpose [6]. Considering broad use of BPA in plastic, we avoided SPE approach. Therefore, we performed liquid-liquid extraction; even this preparation was time-consuming and labor intensive. In a case of biomonitoring environmental phenols, especially BPA, a general problem is background contamination during the analyses, which interfere with quantification at low concentrations of BPA. Therefore, we used glassware throughout the entire analytical procedure in order to avoid possible contamination of BPA. Blank tests, which were conducted with water instead of colostrum, were also performed at every daily experiment to confirm an absence of environmental phenol contamination in the whole of our experimental process. In addition, we tried to our best to avoid a drawback of LC/MS/MS analyses, for example, over estimation in low phenol-contained samples [20]. Thus, we established an optimal condition to measure BPA, OP, and NP in the colostrum samples. We also performed analyses of HPLC/FLD to confirm LC/MS/MS results in low phenols samples (<medians of each phenol: *N* = 50) and obtained high reproducibility between two analyses (CVs < 15%).

Among the present 325 colostrum samples, 70.6% of them have detectable levels of BPA in colostrum (Table 2). Mendonca et al. recently reported similar detection frequency of BPA in USA—breast milk for 3–15 months infants, that is, 75% [21]. However, Völkel et al. detected BPA in 42% of infant urine [22]. Considering the relatively high frequency of BPA detection in colostrum samples, we suggest a high potential for exposure of breast-fed infants to BPA via colostrum. In addition, median level of total BPA (7.8 ng/mL) in the present colostrum study is somewhat higher than those in other biomonitoring studies with breast milk samples [12, 23, 24]. For example, another study with 101 colostrum samples detected BPA at a range of 1–7 ng/mL and a mean level of 3.41 ng/mL [25]. However, they used convenient ELISA methods and detected BPA in all samples without confirmation of BPA free system for analyses, for example, blank tests. Mendonca et al. also reported quite low levels of total BPA (median, 0.8 ng/mL) in small number of infant breast milk samples (*N* = 23) [21]. 

On the basis of three phenol levels in colostrum, volume of daily intakes of milk (500 mL), and mean body weight of the baby subjects (3.24 ± 0.46 kg), we estimated the daily exposure of infants to BPA, OP, and NP (Table 3). The estimated exposure levels of BPA and NP via colostrum in Korean neonates appear to be safe, compared to their TDIs [3, 8]. In a case of OP, we cannot determine whether the present exposure status of OP is acceptable or not at this point because there is no reference dose data of OP. When we compared the daily exposure levels in the present neonate to those in adults, they may be highly exposed to phenols due to body burden. For example, the neonates may be *≈*10-fold highly exposed to BPA than adults [1.2 ug/kg (Table 3)] versus *≈*0.13 ug/kg, which was estimated from Korean adult urines [14]. 

Concerning EDCs' disposition, many researchers have analyzed EDCs at adiposities. As lipophilic characteristics of EDCs can induce chronic diseases even with buffering of acute toxicity, this issue has been emphasized. A recent Spanish study showed that the most obese woman had the highest levels of NP and PCBs in adipose tissue [26]. However, it is not clear, yet, whether body fat contents affect phenol accumulation in the body. In the present study, we found positive associations between levels of mothers BMI before delivery and NP or OP levels. However, BPA levels showed opposite trend, that is, negative association with mothers BMIs. For the reason of the opposite trend, we consider physiochemical difference in octanol-water partition coefficient value (log *P* or Kow) between NP and BPA (4.48 and 3.32, resp.) [27]. In addition, people are simultaneously exposed to multiple EDCs; thus, we screened combined effects of BPA, NP, and OP on the present mothers' and neonates' health. However, the simple sum of total BPA, NP, and OP levels did not show any health risk. In order to study future and real combination effects of phenols or EDCs, their weight for risks or reliable simulation of multiple exposures should be considered.

Referring pharmacokinetics of phenols in human or rhesus monkeys [28, 29], which may be mainly metabolized into urine as conjugated forms with glucuronyl or sulfonyl groups, we have used conjugated BPA for biomonitoring of BPA in urine or blood samples [14, 15, 30]. Detoxification enzymes which metabolize free phenols into conjugated phenols are known to decrease in pregnant women [31]. As most conjugated BPA was analyzed in urine, we analyzed BPA in some urines among the present mothers who donated urine (*N* = 21) to confirm conjugation capacity in the present mothers. Their conjugated BPA levels in urine were approximately half of those in colostrum (mean ± std, 0.98 ± 1.96 ug/L versus 2.44 ± 3.68 ug/L, mean difference, −1.46 ug/L.) When we compared the conjugated BPA levels in the present lactating mothers' urine samples to those in other adults' [14], they were quite lower than the others (median, <0.6 ng/mL versus 7.86 ng/mL). It may support pregnant women' loss of detoxification enzyme activity, even though we consider the decline of BPA exposure due to years [32]. 

We also studied exposure of routes of the phenols in mothers and found association between BPA levels and consumption of dairy products (Table 3). Dairy food always needs its storage, packing, or some manufacturing. The real parts, which people intake, directly contact to containers or packing materials rather than other food. Like PCBs accumulation in perennial fish [3], accumulation of BPA in cow can be thought via food chain. Interestingly, we also found a positive association between BPA levels and use of detergents for food. A recent report [33] concerning the “effect of detergents in the release of bisphenol A from polycarbonate baby bottles” can support our result. Referring reports of AP-related food [16, 17], we studied more other sources for APs, but we could not find any similar source, for example, fish, fruit, or fish oil. 

Considering health risks of phenols, we found negative association between embryonic period in neonates and their colostrum NP levels and suspect NP induces embryonic instability. NP showed some embryotoxicity in crustaceans and oysters, for example, low survival rates and poor embryonic and larval development [34, 35]. However, experimental studies in mammals to clarify effects of NP on embryonic period have been thoroughly performed, yet. Thus, future enlarged epidemiological studies or experimental studies are required to confirm risks of NP on embryonic stability. Secondly, we found that levels of total NP in sick mothers, particularly, toxemia-patients, were higher than those in health subjects (Table 4). In addition, we found that the infant with congenital malformation of uvulas showed quite high levels of total APs in compared to others' levels. Thus, our finding should be further confirmed in enlarged studies, even though the present subjects well represent the Korean women in childbed from similar proportion of toxemia or gestational complications [36]. 

In conclusion, we found that most of neonates are exposed to BPA rather than NP or OP via colostrums. Although current exposure levels of the phenols are safe based upon their TDIs, we suggest continuous biomonitoring of them to clarify their unclear health risk on neonates and pregnant or gestation mothers. 

## Figures and Tables

**Table:** (a) Mothers

Items	% (*N* = 325)
Health status	
Normal	89.2
Disease^a^	10.8

Education	
≤9 yrs	0.3
>9 yrs and ≤12 yrs	22.4
>12 yrs and ≤16 yrs	69.4
>16 yrs	7.9

Job	
House wife	43.0
Office workers	21.7
Teachers	10.8
Others	24.5

Monthly income	
<$2,000	17.1
$2,000~$4,000	55.7
>$4,000	27.2

Food intake	
Vegetable preferred	16.9
Meat preferred	14.7
Fish preferred	14.3
Evenly	53.9

Consumption of dairy products^b^	
1~2 times/month	11.3
Once/week	12.0
2~3 times/week	40.8
Every day	35.9

Use of detergents for food	
Rare	1.0
2~3 times/week	11.2
Every time	87.8

Use of cosmetics	
1-2 times/month	24.1
Once/week	20.6
2~3 times/week	27.3
Every day	28.0

Alcohol drinking^c^	
Never	29.1
1-2 times/month	47.7
Once/week	46.2
≥2 times/week	7.0

Tobacco smoking	
Never smoker	88.07
Ex-smoker^d^	11.58
Smoker	0.35

^a^Toxemia, thyroid disorders, gastritis, and so forth.

^b^Milk, cheese, and so forth.

^c^Before pregnant.

^d^Quitted smoking over 1 year.

**Table:** (b) Infants (*N* = 326)

Items	
Embryonic period (wks)	39.1 ± 1.5
Body weight (kg)	3.2 ± 0.4
Stature (cm)	50.2 ± 2.3
Health status (%)	
Normal	88.7
Disease^a^	5.0
Recovered^b^	6.3

^a^Jaundice (*N* = 6), intestinal obstruction (*N* = 2), congenital malformation (*N* = 2), enteritis (*N* = 1), cyanosis (*N* = 1), and so forth.

^b^Jaundice (*N* = 7), respiratory distress (*N* = 5), conjunctivitis (*N* = 1), dyspepsia (*N* = 1), acute enteritis (*N* = 1), and so forth.

**Table 2:** Comparison between exposure levels and regulation levels of phenols.

	Phenols	Detection of all samples (%)	Median (ng/mL)	Range (ng/mL)	Estimated daily exposure in neonates^a,b^ (*μ*g/kg)	TDI (*μ*g/kg)^c^
Free	BPA	39.8	<LOD	<LOD–54.2		
	NP	0.0	<LOD	<LOD		
	OP	2.9	<LOD	<LOD–14.1		

Total	BPA	70.6	7.8	<LOD–57.3	1.20	50
	NP	15.9	<LOD	<LOD–23.4	0.12	5–15^d^
	OP	27.5	<LOD	<LOD–30.9	0.02	Unknown

Conjugated	BPA	70.6	4.2	0.0–30.2		
	NP	15.9	<LOD	0.0–23.4		
	OP	27.5	0.0	0.0–30.7		

^a^Nondetectible values were designated to half of the lowest value of each phenol.

^b^Daily intake volume of milk in neonates, 500 mL; mean body weight of the infants, 3.24 kg; calculated from median level of each phenol × 500/3.24/1000.

^c^[3, 14].

^d^NOAEL (no observable adverse effect level), 15 mg/kg; uncertainty factor, 1000 (WHO) and 3000 (Danish Institute of Safe and Toxicology).

**Table 3:** Correlations between total phenol levels and candidates of exposure sources.

	BPA		OP		NP	
	Correlation coefficient^a^	*P*	Correlation coefficient	*P *	Correlation coefficient	*P*
Consumption of dairy products	0.36	0.02*	0.04	0.55	−0.08	0.19
Use of detergents	0.12	0.04*	0.07	0.29	−0.06	0.32
Use of cosmetics	−0.05	0.39	−0.05	0.43	−0.04	0.48
Fish preference	−0.09	0.11	−0.00	0.96	−0.02	0.76
Alcohol drinking	0.04	0.46	−0.01	0.82	−0.05	0.45
Tobacco smoking	−0.04	0.46	−0.02	0.78	−0.03	0.67

^a^Spearman's *Rho* by pair wise correlation analyses.

*Statistically significant.

**Table 4:** Association between health status and total phenol levels^a^.

	BPA		NP		OP	
	mean ± std (ng/mL)	*P*	mean ± std (ng/mL)	*P*	mean ± std (ng/mL)	*P*
Mothers (*N*)						
Healthy (290)	7.75 ± 7.36	0.57	2.04 ± 3.71	0.02*	2.24 ± 4.24	0.38
Sick (35)	7.99 ± 7.35		3.51 ± 4.98		2.59 ± 3.66	
Infants^b^						
Healthy (312)^c^	7.83 ± 7.50	0.76	1.86 ± 3.29	0.09	2.20 ± 4.13	0.31
Sick (14)	6.65 ± 7.06		3.95 ± 6.35		3.91 ± 6.06	

^a^Wilcoxon's test.

^b^Data show phenol levels in their mothers' breast milk.

^c^It includes recovered subjects.

*Statistically significant between healthy and sick subjects, *P* < 0.05.
